# “The Less I Think About It, the Better I Feel”: A Thematic Analysis of the Subjective Experience of Malignant Mesothelioma Patients and Their Caregivers

**DOI:** 10.3389/fpsyg.2018.00205

**Published:** 2018-02-20

**Authors:** Fanny Guglielmucci, Isabella G. Franzoi, Michela Bonafede, Francesca V. Borgogno, Federica Grosso, Antonella Granieri

**Affiliations:** ^1^Department of Psychology, University of Turin, Turin, Italy; ^2^Occupational and Environmental Medicine, Epidemiology and Hygiene Department, Italian Workers' Compensation Authority (INAIL), Rome, Italy; ^3^Interdepartmental Functional Unit for Malignant Mesothelioma, SC Oncology, SS Antonio e Biagio e Cesare Arrigo Hospital, Alessandria, Italy; ^4^S Spirito Hospital, Casale Monferrato, Italy

**Keywords:** cancer, malignant mesothelioma, diagnosis, caregivers, interviews, thematic analysis

## Abstract

**Aim:** A cancer diagnosis—and in particular a Malignant Mesothelioma (MM) one—breaks the somatopsychic balance of the individual, compromising the quality of residual life and giving rise to many negative emotions difficult to integrate and to elaborate (such as depression, fears, anxieties, hopelessness, guilt, shame, and rage). Several national and international guidelines acknowledged the importance of evaluating psychological and socio-relational features in MM patients and their caregiver. However, only few studies have investigated the subjective experience of MM patients and even less research has focused on the caregivers' experience. Thus, the aim of the present study is to investigate the lived experience of both MM patients and their caregivers, providing an in-depth comprehension of the psychological sequelae of the disease.

**Materials and Methods:** Within a psychoanalytically-informed conceptual framework, open-ended interviews were conducted with 10 MM patients and 9 caregivers. Thematic analysis was employed: interviews were audio-recorded, transcribed verbatim, and coded in order to identify the main recurring themes across the narratives.

**Results:** We detected four different themes: (1) bodily symptoms and embodied emotions; (2) living in or near a National Priority Contaminated Site (NCPS); (3) “nothing is like it was” (that is, the impact of the diagnosis on everyday life, the changes it causes in the family relationships, the things that are still possible to do, the mourning process); (4) “what will become of us?” (that is, worries about the impact of the diagnosis on the beloved ones, death and legacy).

**Discussion:** MM patients and caregivers seem to be stuck in a concrete mental functioning focused on symptoms and they find it difficult to openly think and talk about the affective and emotional consequences of the diagnosis. Alongside this, they express the need to find new and less conflictual ways to stay together and talk to each other during the period of active treatments for the illness (i.e., chemotherapy, radiotherapy, etc) and the end-of-life. The results of this study have important implications for the clinical management of MM and can help develop multi-professional specialist interventions addressed to both patients and caregivers.

## Introduction

Occupational and environmental exposure to all types of asbestos has become a big concern for public Health Services because of its close causal relationship with several forms of cancer (i.e., cancer of the lung, pharynx, larynx, stomach, colorectum, and ovary; WHO-IARC, 2012). Among those, malignant mesothelioma (MM)—a highly lethal tumor arising from the pleura and, less frequently, from the peritoneal and pericardial serous membranes and from the tunica vaginalis of testis—has been linked almost exclusively with asbestos inhalation. Most recent epidemiological studies report the risk of MM attributable to asbestos exposure to be between 86 and 95% (Rushton et al., 2012; Lacourt et al., 2014).

According to the World Health Organization, Western Countries are currently facing a mesothelioma epidemic, because of the extensive use of asbestos between the 1950's and the 1980's in several industrial applications and the long latency period since the beginning of exposure (WHO, 2006).

Despite medical efforts, advances in treatment are scarcely effective and the MM prognosis remains severe, with an average survival rate of ~9–12 months from diagnosis (Edwards et al., 2000; Lo Iacono et al., 2015; Magnani et al., 2015; Novello et al., 2016), a 2-year survival rate nearly of 50% and a 5-year survival rate of about 20% (Heelan, 2004).

Psychoanalytic literature on cancer describes it as a traumatic condition which threat the somatopsychic unit of the individual, compromising the quality of residual life and giving rise to overwhelming affects, death anxieties, conflicts and fantasies above cancer, life and death that people often try to cope with through defense mechanisms such as omnipotence and denial (Salander and Windahl, 1999; McDougall, 2002; Goldie and Desmarais, 2005). The severity of symptoms and the poor prognosis, along with the awareness of the occupational and “unnatural” origin of the disease and the ethical issues connected to the human responsibilities in the contamination, seem to worsen MM patients and caregivers conditions (Guglielmucci et al., 2014, 2015; Guglielmucci, 2016). They often confront themselves with depressive symptoms and many negative emotions difficult to integrate and elaborate, such as fears, anxieties, hopelessness, guilt, shame, and rage for a terribly unfair diagnosis (Charmaz, 1983; Nowak et al., 2004; O'Brien et al., 2006; Arber and Spencer, 2013; Granieri et al., 2013).

The MM diagnosis does not undermine only the affective life of patients and of those who take care of them, but it also deeply compromises social cohesion and the quality of their external relationships, leading to perceptions of marginalization and withdrawal (Bottomley et al., 2006; Granieri, 2008, 2013; Hughes and Arber, 2008; Borgogno et al., 2015; Zhang et al., 2015).

Despite the importance of evaluating psychological and socio-relational features of MM has been stressed by several national and international guidelines and operational recommendations (BTS Standard of Care Commitee, 2007; Department of Health., 2007; Stahel et al., 2008; Scherpereel et al., 2010; Baas et al., 2015; Granieri, 2015; Novello et al., 2016), these issues still remain understudied and the subjective experience of MM patients and caregivers seem to be rarely taken into account in literature (Moore et al., 2010). Indeed, only five studies have investigated through a qualitative approach patients' subjective experience of living with MM, highlighting the need for a more in-depth understanding of their distinctive emotional suffering and psychological needs, advising future research on the topic (for a systematic review see Ball et al., 2016). For example, Arber and Spencer (2013) explored patients' experience during the first 3 months following the diagnosis: they pointed out that the uncertainty and lack of control connected to the illness lead to a great emotional, physical and psychosocial distress, as well as to worried about the fast progression of the disease. Similarly, Clayson et al. (2005) research showed that MM patients experience high symptom burden, worsen by distressing medical treatments that cannot lead to a recovery in any case. They tended to deal with MM and its consequences with a stoic attitude, relying heavily on their first-degree caregivers.

As regards, the subjective experience of caregivers is even less considered: indeed, only one study has included an in-depth investigation of their lived experience, underlining the need for supportive care to improve quality of life also in family members during the treatment trajectory. Moreover, it has highlighted the need for a careful attention during the legal requirements after death, such as coroner's inquests, that can increase the psychological suffering of the caregivers, if they are not supported by trained professionals (Hawley et al., 2004).

As far as we know, no study in Italy has assessed the subjective experience of MM patients and their caregivers through qualitative methods. Thus, the aim of the present study is to explore the subjective impact of the MM diagnosis on both patients and their caregivers, implementing previous qualitative researches in the field.

## Materials and methods

The above research questions were addressed through an explorative qualitative research design, focusing on audio-recorded open-ended interviews of 10 MM patients and 9 of their first-degree caregivers. Within a psychoanalytic approach, data were analyzed through thematic analysis to draw out individuals' experiences and meanings. We decided to rely on an inductive data-drive form of thematic analysis, a bottom-up approach where data collected informed—instead of being informed by—theoretical considerations (Glaser and Strauss, 1967; Charmaz, 2006).

Two trained clinical psychologist carried out the interviews, which were independently coded by three of the authors (IGF, FaG, and MB) following Braun and Clarke's six step method (Braun and Clarke, 2006).

1. Familiarizing with data: checking the accuracy of the transcriptions and generating an initial list of interesting segments across the data set.
2. Generating initial codes: identifying semantic contents useful to explore the lived experiences of participants and manually coding them. Progressively emerging codes were compared to existing ones, similarities and differences were evaluated and new codes were created and applied to previous transcripts (Patton, 2002).
3. Searching for themes: performing a combination of codes to create overarching themes and building mind-maps to represent relationship between codes, themes, and different levels of themes. Relationships between codes and themes were developed through axial coding until each category was refined and saturated (Strauss and Corbin, 1998).
4. Reviewing themes: discussing identified themes and grouping them into clusters, comparing data-extracts across participants and highlighting illustrative examples. All authors were involved in this discussion and only the themes with an overall consensus were maintained.
5. Defining and naming themes: discussing the definition and the name of each theme and sub-theme until an overall consensus was found.
6. Producing the report.

This method has already been used in several studies in the last decade (see among others: Malik and Coulson, 2008; Hawkins et al., 2009; Hussain and Griffiths, 2009; Smith et al., 2009; Brennan et al., 2010; Frisén and Holmqvist, 2010; De Ganck and Vanheule, 2015; Crowe et al., 2017; Fullwood et al., 2017; Larkings et al., 2017; Soilemezi et al., 2017). In line with the above-mentioned literature, we decide to not quantify our qualitative data.

Research was carried out in Casale Monferrato (Italy), a National Priority Contaminated Site (NPCSs). NPCSs are areas of great concern for remediation, most of them with a long-lasting industrial activity, and with a high incidence of several cancers, connected to the presence of different carcinogenic pollutants (Comba et al., 2014). An epidemiological survey conducted within the SENTIERI Project together with the National Register of Mesothelioma (ReNAM) on 39 NPCSs with a high incidence of MM clusterized Casale Monferrato in the higher risk group, with an incidence of MM of 68.6 × 10.000 × year (IC90% 62.6–75.1) in males and 45.8 × 10.000 × year in females (IC90% 41.1–50.8; Binazzi et al., 2016). Moreover, Casale Monferrato represents a peculiar reality among NPCSs, because pollution is connected to an individual pathogen and a distinct pathogenic source (the Eternit factory, which ceases its activities in 1986). Casale Monferrato cluster is characterized by a high proportion of cases with environmental exposure (above 15%) and high number of MM cases occurring in women (Corfiati et al., 2015).

The present study is part of the AVPM Project (Assessment delle Variabili Psicologiche nei casi di Mesotelioma—Assessing Psychological Variables in Mesothelioma), which involves the National Health Center for Asbestos, the Interdepartmental Functional Unit for Malignant Mesothelioma (UFIM) of the SS. Antonio e Biagio e Cesare Arrigo and Santo Spirito Hospitals, and the Post-Graduated School in Clinical Psychology (SSPC) of the University of Turin (CCM protocol number J19E12001060001). All subjects participated anonymously in the study and gave their informed written consent. The AVPM Project, including the present study, was approved by the local Institutional Review Board (IRB) and the Hospital Ethical Committee (AVPM-14/11/2014), and was conducted in accordance with the Helsinki declaration. All the participants' first names has been are assigned names given to protect the real names of the subjects involved.

### Interview process

Data were collected through open-ended audio-recorded interviews focused on how patients and caregivers experienced the impact of MM diagnosis in everyday life and family relationships, and on how they lived the subsequent treatments. The interviews lasted on average 45 min, with a duration range between 30 and 70 min. They took place in a reserved room for the consultations at the UFIM.

Key areas of the interviews were:

1. asbestos exposure and work history;
2. treatments history;
3. subjective impact of the disease on the individuals and on their relationships, particularly the family ones;
4. strategies to deal with end of life, death, and survival.

When participants did not talk about the prognosis of the illness, no explicit questions were made about dying, but plans for the future were alike explored. Patients and caregivers were interviewed together in order to promote communication between family members on MM diagnosis and its impact.

### Participants

Participants were consecutively recruited and interviewed between October 2014 and January 2015 at the UFIM.

Inclusion criteria were:

1. having a diagnosis of MM in any localization;
2. having the diagnosis during the recruitment period;
3. signing an informed written consent form.

Exclusion criteria were:

1. having a poor knowledge of the Italian language;
2. having a certified psychiatric diagnosis;
3. having a certified diagnosis of a neurogenerative disease (i.e., Alzheimer disease, Parkinson disease, etc.).

During the recruitment period, 21 new diagnosis of MM occurred, consistently with epidemiological data for Casale Monferrato (Ministry of Health, 2012). Three (14.29%) subjects were excluded because of their critical clinical conditions, 2 (9.52%) chose to be followed in another District, 6 (28.57%) decided not to give their consent to the interviews, thus they did not take part in the study.

The final sample (*N* = 19) is composed of 10 patients (52.63%) and 9 caregivers (47.37%). Table 1 shows the socio-demographical and clinical characteristics of the sample, differentiated for clinical condition (patients/caregivers).

**Table 1:** Socio-demographic and clinical characteristics of the sample.

	**Full sample (*****N*** = **19)**		**Patients (*****N*** = **10)**		**Caregivers (*****N*** = **9)**	
	***M***	***SD***	**Range**	***M***	***SD***	***M***	***SD***
**Age**	61.53	11.49	46–77	62.60	10.47	60.33	13.06
	***N***	**%**		***n***	**%**	***n***	**%**
**Gender**							
Males	8	42.11		6	60.00	2	22.22
Females	11	57.89		4	40.00	7	77.78
**Educational Level**							
Elementary school	1	5.26		0	0.00	1	11.11
Middle school	8	42.11		6	60.00	2	22.22
High school	5	26.32		1	10.00	4	44.44
Degree	3	15.79		2	20.00	1	11.11
Master's degree	2	10.53		1	10.00	1	11.11
**Work**							
Employee	12	63.16		6	60.00	6	66.67
Teachers	2	10.53		1	10.00	1	11.11
Freelance professionals	2	10.53		1	10.00	1	11.11
Laborer	1	5.26		1	10.00	0	0.00
Retired	2	10.53		1	10.00	1	11.11
**Family Status**							
Married	17			9	90.00	8	88.89
Cohabitants	2			1	10.00	1	11.11
**Diagnosis**							
Biphasic pleural mesothelioma				3	30.00		
Epithelioid pleural mesothelioma				5	50.00		
Sarcomatoid pleural mesothelioma				1	10.00		
Peritoneal mesothelioma				1	10.00		

## Results

**Figure 1:**
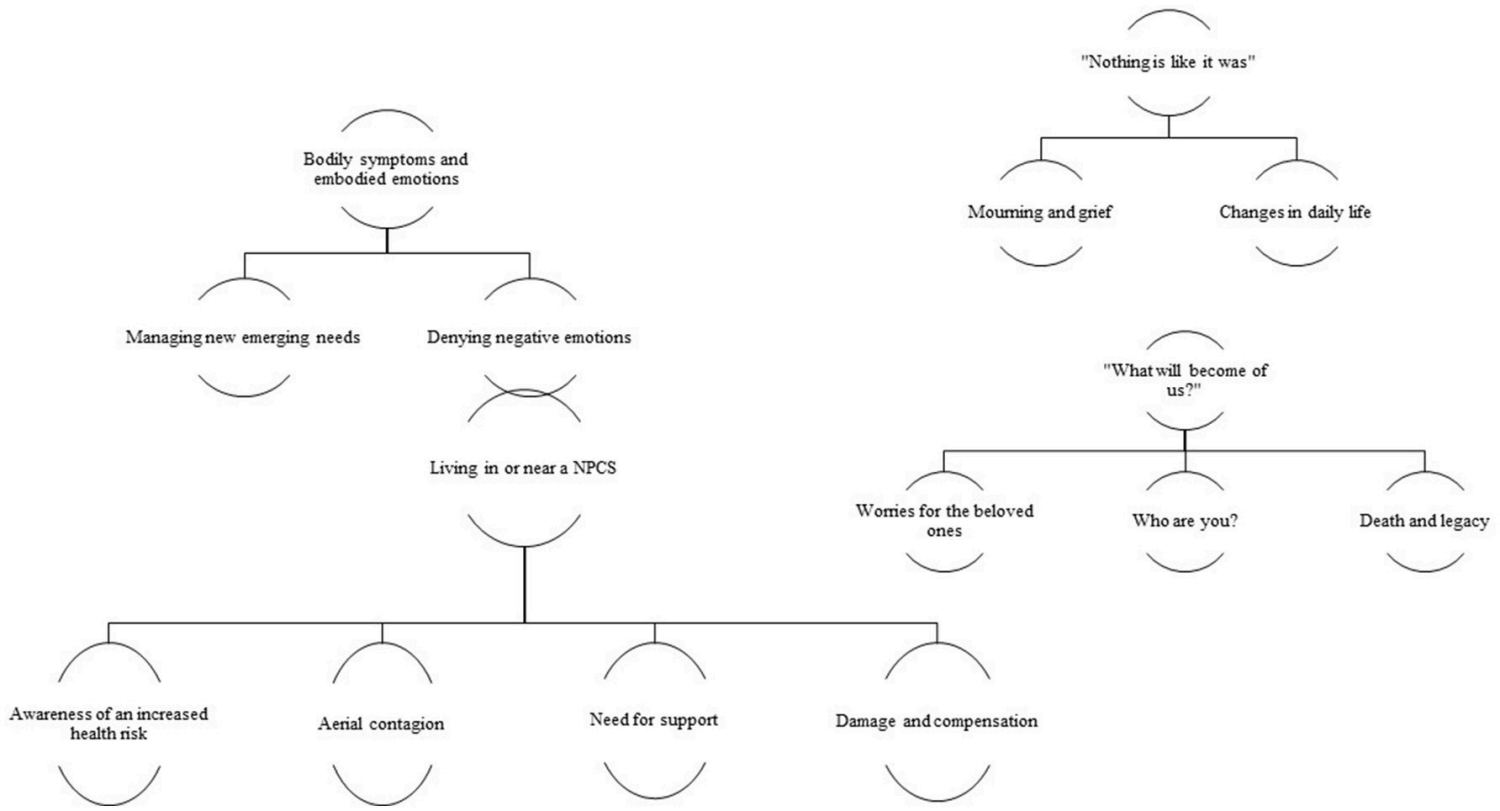
Thematic map.

Thematic analysis identified four different themes: bodily symptoms and embodied emotions; living in or near a NPCS; “nothing is like it was”; “what will become of us?” (Figure 1).

### Bodily symptoms and embodied emotions

#### Managing new emerging needs

Eight of 10 MM patients left free to speak about their illness started their accounts talking about their medical history and the physical facets of the disease: the first symptoms, how they discovered to have MM, their medical treatments (i.e., chemotherapy, radiotherapy, surgery), and the experimental trials they were attending. Many patients' first symptoms were connected to eating or drinking.

Attilio (patient): We were at the restaurant, we were having ravioli […] and they had a weird taste.

Elisa (patient): I couldn't [taste] anything no more. I couldn't drink water anymore.

The remaining two patients told they did not notice any physical changes until late.

Clara (patient): I didn't notice anything. And it was already at an advanced stage […] If I had had some clue. Maybe there was, but I didn't saw nor felt anything.

The medical journey to identify the causes of such symptoms—to give them a name—had been often long, winding and frustrating.

Rosangela (patient): We had already done the x-ray, but we did it again, and once again it wasn't possible to see anything, […] So she [her doctor] started to tell me: “try and go to the osteopath, maybe …” as they told me that it was an adhesive pleurisy […] so I've done some culturing and now I can't remember what kind it was […] and I went to the gym, then I went to the osteopath [then to the physiatrist, who told me] “try to go swimming”. […] then I went to my doctor again and he told me: “come on, […] we have to do some blood tests anyway”. Then he saw I was crying [and he told me]: “try and go to [the Chief of Internal Medicine Department]”. They called me and told me: “come” and they visited me and told me right way “go and take the [CAT scan]”. […] I almost went through everything.

The description of the invalidating symptoms and treatments was often factual and concrete, with sometimes a small space for emotions connected to the diagnosis. The impact of MM was mostly narrated talking about the physical changes it caused and the difficulties to accept that.

Luigi (patient): I mean, now I had a little bit of… I was quite worried because I had this cough, this thing. But well… this cough. She [the oncologist] told me: “look, you're alright. It's just a cold, nothing more than a cold, you're feeling great.

Elisa (patient): You can't clean the bathrooms, the kitchens, the floo… the windows. Uff. It's just absurd! You physically can't. […] Before I was used to be autonomous, indep… while now you always have to depend on others.

Fabio (patient): You become quite impeded, that's it. Quite… quite a lot.

Rosangela (patient): I hadn't the strength to walk, to stand. Even if someone tells you: “you've got to make an effort, you've got to make an effort”. But what if I really can't?

Moreover, during the interview process a large part of patients (7/10) were involved in clinical trials at the UFIM, a high-specialized Functional Unit for MM treatment, underlying their sensations to be like “guinea pigs.”

Clara (patient): Let's see, we'll see. We'll get to see where those treatments will take us. I'm like a guinea pig, we'll see. Maybe well… We'll carry this on, I don't know, because it's a clinical trial.

Elisa (patient): We are all like guinea pigs. It socks but it must be like that: until the oncologists find a cure, they have to use guinea pigs.

#### Denying negative emotions

For both patients and caregivers getting in touch with the emotional impact of the illness seem to be very difficult and they appear not to be able to put into words the intense feelings of shame, guilt and blame connected to the failing health status. For a large part of them it seems to be easier an emotional and social withdrawn, taking on an “out of sight, out of mind” attitude.

Rosangela (patient): Well, I wasn't able to go to church anymore. Not exactly to church, to the mass in my town, because I didn't want to face anyone, even now […] because: “how are you feeling?” And “how are you doing?”. They ask you a lot of questions, let's say. I've changed, I had to change my church.

Fabrizia (caregiver): Unfortunately, people are ignorant, because it happened to us that we were at the supermarket and we met some people that looked the other way. […] And they asked her [the mother, who has the MM] if everything was ok, but then that was it.

Anna Rita (caregiver): There's nothing to be ashamed of, huh. I think. Because there are people who really […] don't talk about that, you know. […] What do you want to hide anyway?

Rino (patient): [and they ask you]: “How are you doing?”. How do you think I'm doing? Don't be a pain in the ass!

Bruno (patient): [another patient] told me: “why are you so low? You don't have to think about that. I never think about that”.

### Living in or near a NPCS

#### Awareness of an increased health risk

During the interviews, all the subjects talked about the etiology of MM connecting it with the geographical area where they were used to reside. They were all aware that living in or near Casale Monferrato has represented since their childhood a high risk for developing MM and that it could still represent a risk for their health and the health of their beloved ones.

Attilio (patient): Obviously, as a child I breath a great amount of dust because we went to the Po river. We cycled along that… where there was that enormous pipe with its dust emissions that came out from time to time and so on. But we closed our eyes and we kept on cycling because otherwise we would have ended up in the canal. So, we went straight forward, but we inhaled something as now I suffer the consequences. I wouldn't believe that after 47 years, you know, this would come out.

Clara (patient): This really touched me. This can happen to anyone, because it's not about smoking, that you can say: “Well, I deserved it.” It's something that just happened, it happens to anyone [here], it could happen to anyone.

Mauro (caregiver): Unfortunately, [it happens] around us every week, you even read about this on the newspapers.

Elvira (caregiver): Asbestos doesn't forgive.

#### Aerial contagion

Beside the denial of the negative emotional impact of MM, the fear for an “aerial contagion” of the disease could be traced in many participants' words. Some patients and caregivers talked about their feeling of being pointed at as some sort of “plague spreaders” by the community.

Mirella (caregiver): I mean, he [her husband, who has MM] doesn't have the leper.

Death anxieties and a sort of “phantom of death” that could strike everybody, even the strongest ones, seem to arise from their narratives.

Claudio (patient): Many friends of mine died because of mesothelioma. This makes you wonder who will be the next one.

Gabriella (caregiver): And there was a lady back in those days, in that very week when I want there, there was a lady who passed away that very morning. […] It was harsh; I still remember that. […] Because she probably was at the very end. We were there for the hydration. […] She was a lady I knew, she had the strongest body that lady.

#### Need for support

Some patients and caregivers put into words their need to find someone whom they could share with their illness experience. Preferably someone outside the family, someone who was living the same experience or who could give them emotional support and comprehension.

Marta (caregiver): I count very much on […] the possibility that he [the husband, who has MM] can compare himself to others with the same disease. So that he can stop feeling as he was marked by God. Because he says very often: “Why is this happening to me”?

Clara (patient): Well, yes. I think talking can be useful sometimes, uh. Sometimes it's enough! Talking with friends that are open to… maybe. […] With the ladies I know. [And] I have sisters that really cooperate: I'm lucky!

Alongside this, the need to find new ways to talk with their relatives also emerged.

Rosangela (patient): He [her husband] doesn't want to talk about my disease. […] He doesn't talk with anybody. […] He is annoyed, because he says that this is our business. [And I tell him] that I know that's our business, but we cannot do anything about it. We didn't bring this on ourselves!

Mirella (caregiver): Maybe [talking] in one way or another you can exchange views and ways to manage all of these, that could be completely different.

#### Damage and compensation

Almost two-thirds of the participants (12/19) talked about the Eternit factory, where asbestos was produced for decades, and the legal battle for the compensation process.

Fabrizia (caregiver): They actually found mesothelioma cases caused by the kind of jobs they did inside the establishment. We pressed charges, I mean a report was filed and all. And now at the end of December they told us that they didn't recognize [the damage for her]. The Trade Union itself suggested to appeal. […] And so maybe even this at a psychological level, let's say, insult on top of injury. Because […] you ran everywhere, they wanted witnesses, you had to go for checkups and visits, and on top of that in the end we got a negative outcome.

Rino (patient): To attest that it is an occupational disease, I had to go to the INAIL and take some witnesses. They went there and answer all their questions: if it was true that I worked there, what did I do for work, which materials I used. They make it harder so that they don't have to pay: after all, it's mesothelioma. You should have the right and stop.

Bruno (patient): They asked me to give them my occupational card and everything came out. Because I've worked at the X since it was closed and then I've worked at the Y, where I brought the concrete to the Eternit. I worked in the middle of dust, there. Then for years I've done other jobs, but asbestos was already there.

### “Nothing is like it was”

#### Mourning and grief

Both patients and caregivers seemed to be engaged, in different ways, in the process of mourning about health and life as it could be. Some of them showed avoidant attitudes toward the emotional burden connected to the awareness of the MM poor prognosis, others showed a mood deflection, while in some cases thinking about the disease and the approaching of death gave rise to terrifying and paralyzing emotions linked with the perception of one's own finitude and mortality.

Luigi (patient): There are times when you just, uh, you just mope around up.

Elisa (patient): The problem is that you become too terrified.

Clara (patient): It isn't easy; you know? It isn't easy for anybody. What am I supposed to say? I really don't know what I could say. You start living for today.

Rosangela (patient): It's stronger than me having these dark thoughts […] I'm always scared. […] And so many times I wake up in the morning and I think: “Uh, I woke up!”

Luisa (caregiver): Let's say that I've tried not to think much about this, but I'm falling down, I realize, in a sort of depressive mood.

Elvira (caregiver): Well, I take it badly, but really… very badly.

Gabriella (caregiver): I've started feeling blue, and bluer, and bluer.

Antonio (caregiver): We're all upset.

Fabrizia (caregiver): In 10 days, let's say, our lives, and most of all her life, turned upside down.

Mauro (caregiver): You try not to think about it. The less I think about it, the better I feel.

#### Changes in daily life

Patients focused on how their lives had changed because of MM, sometimes minimizing the impact of the diagnosis, sometimes putting emphasis on making the best use of the time left, sometimes talking about small goals they desired to reach and things they would like to do.

Fabio (patient): Well, nothing is like it was. You have to think about your situation and make it go on in another way.

Rino (patient): You have to take it like it is. How can you prevent yourself from thinking about it? But you don't have to [feel so down]. It only hurts more! What can you do? That's what happened.

Silvio (patient): You try to look forward.

Bruno (patient): You have to take life as it is. […] Work up the courage and go on. […] I don't see another way. […] These things happen: that's life. If you try and react, you can live well enough that route you still have to work over, while if you stay there just feeling sorry for yourself you worsen everything. You have to be your own psychologist.

Claudio (patient): Well, I think that nobody can have, can foresee the future. You can see some little things, but not [everything]. So, I feel that I can go on. That I can, let's say, not defeat it, but psychologically live all this.

### “What will become of us?”

#### Worries for the beloved ones

Patients more often than caregivers expressed worries about the impact of the diagnosis on their relatives.

Elisa (patient): Well, I think I can handle it, but what about him?

Rino (patient): Well, in just few words she is more touched than I am.

Bruno (patient): If you take it that way, if you take it out, it's worse. You are hurting yourself.

Rosangela (patient): Well, there's the one who has MM who is having a really hard time with this, but there is also the family. The family just cannot accept that. […] And it's more difficult for them because they cannot know what the patient is feeling, what he has inside.

Fabrizia (caregiver): A thing that really strikes me is that she isn't optimistic in saying: […] “you see? Everything is gonna be alright.” Yet, she says: “no,” after all maybe that's because this disease is her only though, at this point.

#### Who are you?

Caregivers instead tended to aggressively underline that their relatives changed after the MM diagnosis, grumbling against them and complaining they were no more as they used to be.

Elvira (caregiver): It's like living as rats hiding in the hole. […] Why isn't he playing [the saxophone] anymore? Why is he behaving like that? Why doesn't he have these things [he used to do]? I mean, I try not to show that, but thinking to myself I always think: what the hell! Before, his feet were always cold, now they're not. Of course, I'm happy, but I think: there's something wrong with that!

Gabriella (caregiver): He isn't him anymore.

Luisa (caregiver): They have always told him that he can live a normal life. He is the one who refuses to do so. […] He could live a very normal life, but he doesn't, because he knows he is sick. […] It makes me even madder that I cannot convince him to have a normal life. Well, I understand very well that's really hard having to face an enemy such as the mesothelioma, uh? […] But you need to have the strength to say: it won't beat me, I'll beat it.

Such communications had a huge negative impact on patients, triggering a circuit of aggressiveness and withdrawal that impairs family exchanges.

Bruno (patient): She hurts herself and she becomes annoying with me.

Rosangela (patient): It's not that I don't want to fight or that they bother me. Well, but I'm saying that they always poke me.

Rino (patient): You have to stop bothering me. It's not that I am mean and I don't want to do that. I just cannot anymore!

#### Death and legacy

Only two patients (Bruno and Rosangela) were able to imagine the life of their beloved ones after their death and to talk, more or less explicitly, about their worries for the survivors.

Bruno (patient): The point is that, if you go there, if 1 day I… when I won't… I won't… If it worsens, I don't know when, and you arrive at this point already emotionally broken, you won't be able to… to do anything. If on the contrary you are able to deal with it without making a drama of it, well, things will be easier.

Rosangela (patient): Yea, because sometimes when he refuses to learn how to cook I say: “And when I'll be no more here, what will you do?”. When I'll come to an end, you still have to eat. You have to cook yourself, uh? Who's gonna cooking for you? You'll call a maid?

## Discussion

The aim of our research was to understand within a psychoanalytically-informed conceptual framework the subjective impact of living with a MM diagnosis in both patients and their caregivers. Through a data-driven thematic analysis we identified four different common themes: bodily symptoms and embodied emotions; living in or near a NPCS; “nothing is like it was”; “what will become of us?”

The largest number of patients' and caregivers' narratives focused on the medical journey to identify first symptoms' causes, and on the physical limitations caused by the disease. Participants seem to speak easily about their sick body, the severity of their medical conditions and treatments they are attending to. Unlike in other realities (British Lung Foundation, 2007; Mesothelioma UK., 2012), in Casale Monferrato both patients and caregivers seem to be aware of MM etiology, its consequences, and the steps needed be followed during medical interventions. Probably such increased awareness of the health risk for MM and the understanding of the medical aspects of the illness is linked with living in a NPCS, an area characterized by a great media resonance because of MM's incidence and by the strong role of the Association of Families and Asbestos' Victims (AFeVA), which has led in years to a large presence of prevention and sensibilization campaigns (Granieri, 2016a,b).

For both patients and caregivers talking about affects connected to MM diagnosis and its prognosis is something very difficult to manage: emotions seem to be avoided and participants appear more able to produce a factual description of symptoms, stressing the restricting impact of MM on everyday life. Indeed, as the Italian psychoanalyst Franco Fornari (Fornari, 1985) pointed out, in cancer patients affects have often been buried by the disease. After the diagnosis people start feeling hopeless and unable to elaborate and symbolize affects connected to the illness. Nevertheless, beneath this difficulty in producing symbols and the tendency to speak through symptoms, a deep trace of shame, guilt and blame can still be traced. For example, Rosangela, a MM patient, argues she does not want to face anyone who knows about her illness because they would ask too many questions, which would imply thinking about too many painful answers and the negative emotions linked with them. In our opinion, these difficulties in integrating emotional states connected to MM into a consistent system of meanings are related to a lack of affect regulation. According to neuropsychoanalytic perspectives, repeated traumatic environmental stimuli—such as the inhalation of asbestos for several years because of environmental and occupational exposure, the loss of many family members and friends, being victims and/or witnesses of a collective trauma that has damaged an entire community—impair the subjective capability to develop mature strategies to represent and regulate affects and emotions, which remain embodied, potentially evoked and related with behaviors (Schore, 2001, 2011; Schore and Schore, 2014). “*Wherever words remain smothered, most of the time we are faced with vague and anguished mental atmospheres. In such states, patients react to deep psychological suffering until they reach an archaic mental functioning in which they do not make use of language, but of psychosomatic expressions*” (Granieri, 2011, p. viii).

These implicit traumatic memories are the result of a constructive-reconstructive process influenced by the historically first modalities to deal with traumatic circumstances, which define the future ways the subject relies on to categorize new analogous circumstances (Leuzinger-Bohleber, 2008; Gallese, 2015). Patients are often told not to think about their illness and its consequences, a sort of “you don't have to think about that” attitude that could have been traced in Casale Monferrato since the Eternit factory started its activities (Granieri, 2016b). As Mauro, a MM patient, pointed out: “You try not to think about it. The less I think about it, the better I feel.”

Thus, the citizens' difficulties in openly speaking about affective and emotional consequences of MM could be attributed to the introjection of a cultural paradigm and the latter recategorization processes connected to the original traumatic experinece of being part of a community highly exposed to death because of the enrichment of some few. Both MM patients and caregivers seemed to collude with this implicit imperative, thus revealing the tendency for denying mortality and avoiding the reality of a deadly illness (Lebovits et al., 1983; Arber and Spencer, 2013). As Straker (2013) pointed out, these mental processes are typically observed in cancer patients, who often find themselves alone in front of cancer and overwhelming death anxieties they try to cope with through the activation of primitive defenses (i.e., denial, splitting, omnipotence). A number of studies on other fatal diseases, such as amyotrophic lateral sclerosis underlined denial as a common coping strategy in patients (Kleinbub et al., 2015; Cipolletta et al., 2017). A similar result has been showed in chronic illnesses (Ketterer et al., 2004; Telford et al., 2006; Marlow et al., 2016). However, the clinical work with chronic illnesses patients and with MM patients is different. Indeed, in the first case it implies mentalizing the necessity to live for life with a modified body and a compromised state of health. On the contrary, MM patients have to deal with limitations connected to the perspective of death in the near future and with a shortened time to share with their beloved ones.

Dealing with MM could be a too harsh reality to face and it could be easier for some patients and caregivers pretending not to see. In fact, for many participants thinking about death and emotions “moved” by MM diagnosis is something to be avoided like plague and some of them feel totally stunned and flooded by shame, guilt and rage. The silencing and distancing community reactions to MM make some patients feel like some sort of “plague spreaders”: only few of them firmly oppose this attitude, claiming they do not have leper, they are not untouchable, and they are not contagious. These words reveal, at the same time, the patients' need for human contact and the citizens' fear of an “aerial contagion,” which seems to impair social relationships and give rise to rage and unconscious death anxieties within the community (Granieri and Borgogno, 2014; Borgogno et al., 2015; Granieri, 2017).

Some authors have described cancer as a “social stigma,” which seems to entail also hospital staff. For example, Goldie and Desmarais (2005) suggested that “*cancer is feared inordinately in our society and its diagnosis is a social stigma that affects both the sufferer's self-perception and the perception of the patient by others. It has common ground with leprosy in that many people are afraid that cancer is ‘catching’* ” (Goldie and Desmarais, 2005, p. 27)

Exploring cancer from a psychoanalytic perspective implies thinking about affects and relational internal/external life, considering negative thoughts, fantasies, and emotions about the past, the present and the future (Chiozza, 1981, 1986). Cancer patients and their caregivers are at the same time helpless victims and witnesses of a tragic reality painful to face: in need of cares and stuck in an enemy body the former, rescuers eager to offer help and reassurances (even concrete) the latter (Fornari, 1985). MM patients and their caregivers find themselves locked in a traumatic condition they are not able to escape, overwhelmed by intense feelings they are not able to regulate and without the possibility of controlling their own body, health, and entire life (Lebovits and Strain, 1984; Lebovits, 1985; Clayson et al., 2005; Hughes and Arber, 2008; Arber and Spencer, 2013).

This affective pattern has been labeled “Damocle Syndrome” (de Villiers et al., 1997), a limbo characterized by a great unpredictability and confusion on what is going to happen next in the treatment trajectory and in the ongoing existence of a cancer patient. It is possible that such syndrome is even more detectable in MM patients and caregivers because of the absence of effective treatments and defined guidelines for this kind of disease. This condition is something hard to face, which deeply weaken patients' and caregivers' safety, as well as their trust in health professionals and in medical interventions. Indeed, many patients of our sample involved in clinical trials refer their sensation of being and being used as a sort of “guinea pigs,” thus underlying aggressive reactions toward institutions. Actually, being part of a trial is strictly connected with important questions and conflictual emotions about the effectiveness of the treatments and/or the possibility of having placebo, compromising the doctor-patient relationship (Straker, 1998). This is a key feature to consider in both in clinical trials and clinical work with cancer patients, because of its possible consequences on patients' compliance and trust in Institutions (Granieri, 2015).

As other cancers, MM does not affect only the ills, but it also changes the shape of their relationships with their beloved ones, giving rise to the difficult question: what will become of us? Both MM patients and caregivers feel deep crisis and mourning because former actions and lives are now precluded by the illness and they need to revise hopes and expectations knowing that they have a short time left to spend together (Charmaz, 1983; Rodin and Zimmermann, 2008). It has been suggested that physical, identity and relational changes caused by MM promote a sense of frustration and helplessness in patients and caregivers that generated emotional distance, and deeply compromise the quality of family exchanges (Hawley et al., 2004; Clayson et al., 2005; Hughes and Arber, 2008). In our sample, caregivers tend to destructively underline that their relatives have changed after the MM diagnosis, and that they are no more as they were used to be. Such communications are deeply harmful for patients and the disease seems to become a sort of threat for the bond, triggering a circuit of anguish, aggressiveness and withdrawal (Franzoi et al., 2015, 2016; Granieri, 2016a).

The relational exchanges within the family must be handled-with-care and talking about the affective meaning of MM in front of the beloved ones seems to be very difficult. Indeed, it does not surprise that participants refer a strong need to find someone—preferably outside the family—whom they could talk to about their hard and painful illness journey. We could hypothesize that such a conflictual and distancing climate is something that impairs the emotional and verbal exchanges within the family and the capability of sharing the illness experiences at home. Specifically, caregivers seem to have grater difficulties than patients in openly verbalizing their present worries for their beloved ones. Probably, this finding can be explained considering the big rage of caregivers for all the changes that the illness has caused in their beloved ones, as we have suggested above. Thus, the aggressiveness and the rage for an unfair fate and future seem to occupy their entire internal world and they are not able to stay in a more depressive position, which implies getting in touch with their vulnerable, helplessness and needy inner parts and making a start on the mourning process (Guglielmucci, 2016; Granieri, 2017).

Patients express their worries about the present impact of the diagnosis on their beloved more frequently than their concerns about what will happen to their caregivers when they will not be here anymore. Only two of them (Bruno and Rosangela) openly talk about their preoccupations for the life of their beloved ones after their death. From a psychoanalytic perspective, this could be connected to the fact that patients know that, in a sense, they are not going to leave their beloved ones, but they are already leaving them, because they will never stay together as they were used to before the illness. Patients are interested in evaluating if their caregivers are able to face their leaving in the present because it is in the present that they need to start loosing their relational bonds so that they can have more resources for themselves, for interacting with the upcoming reality of ill persons close to the end of their lives (Granieri, 2017).

Nevertheless, we can trace in patients' and caregivers' narratives the need to find new ways to talk to each other and maybe a more adaptive way to face and put into words separation and death anxieties (Straker, 2013). From a clinical point of view, our choice to interview together patients and caregivers has turned out to be useful for both patients and caregivers, because it has promoted a new space and way to communicate between family members. Indeed, the presence of a trained psychologist has fostered the possibility to think—often for the first time—about unintegrated mental states, giving them a name and sharing them in a less conflictual way. We believe the presence of a trained psychologist could be very helpful also during the diagnosis' communication, a task that gives rise to intense death anxieties even in health professionals. It does not surprise that they often report the diagnosis communication as one of the most traumatic and stressful features of their work (Vandekieft, 2001; Hagerty et al., 2005), so that in clinical practice the MM diagnosis is often communicated only to the caregiver.

Our findings have important implications for the clinical management of MM patients and suggest the importance of considering the emotional features of MM and the impact of the diagnosis on both the ill person and his/her caregivers, as advocated by national and international guidelines. In our opinion, it is fundamental to develop interventions aimed at increasing the possibility of thinking about MM diagnosis and its multidimensional impact, verbalize and mentalize negative emotional unintegrated states, which remain “operative” in an unconscious part of the Self, negatively compromising the residual life of patients and caregivers. A pivotal step in this process is training clinicians so that they can cope with their own death anxieties when they are facing patients' and caregivers' ones. An adequate training is also needed to support every tiny ray of vitality and to foster a “perspectival vision” (Borgogno, 2014), helping patients and caregivers moving up from a rage-helplessness circuit and a concrete mental functioning to a more symbolic and flexible one.

## Limitations

Qualitative methods cannot provide statistically representative data, nevertheless they provide very important information, which could help clinicians obtain an in-depth comprehension of the lived experience of people suffering from cancer and of those strictly involved in the process of cure/care.

This study is limited by a small sample size, even if recruitment problems in terminally ill patients are well-documented in literature, even more with a rare cancer such as MM (Dean and McClement, 2002). Furthermore, we are aware that patients and caregivers with most severe symptoms or psychological suffering may have not accepted to be interviewed. It could then be stated that all participants live in or near a NPCS and no patient was in the end of life phase, thus data could be not generalizable to patients in other phases of the illness or to people who live in towns where asbestos exposure has not a community dimension and health care services are less prepared to deal with such pathology. Conversely, we believe this point could also represent a possible strength of the present study because it provides a qualitative perspective on a NPCS, describing the approach of the Italian National Center for Asbestos in such a critical situation.

## Future directions

We strongly believe the need to develop health care services for NPCS and tailored interventions to ameliorate patients' and caregivers' psychological wellbeing in these circumstances. In line with this, an in-depth comprehension of the lived experience of patients and caregivers can play a pivotal role in the process of building new efficacy multidisciplinary interventions and identifying specific protocols. Indeed, increasing our sample size we could apply our results in improving the quality of the psychological support for MM patients and their caregivers, that should be focused on the peculiar common areas of suffering as highlighted through the thematic analysis of the interviews. Policy solutions that take into account these aspects could look toward the achievement of an early identification and treatment of anxiety, depression, or burden. Moreover, since the experience of MM is traumatic for both the patient and their caregivers, offering an early support for all those involved could decrease the risk for developing post-traumatic sequelae.

## Author contributions

FaG and IF: Contributed to the study design, data analysis and interpretation, drafting and critical revision of the manuscript; MB: Contributed to the analysis and interpretation of data, drafting and critical revision of the manuscript; FB and FeG: Contributed to the interpretation of data and drafting the manuscript; AG: Contributed to interpretation of data, giving an important clinical and intellectual contribution; All authors approve the final version of the paper to be published and agree to be accountable for all aspects of the work in ensuring that questions related to the accuracy or integrity of any part of the work are appropriately investigated and resolved.

### Conflict of interest statement

The authors declare that the research was conducted in the absence of any commercial or financial relationships that could be construed as a potential conflict of interest.
